# Kinetic modeling and parameter estimation of TSPO PET imaging in the human brain

**DOI:** 10.1007/s00259-021-05248-9

**Published:** 2021-03-11

**Authors:** Catriona Wimberley, Sonia Lavisse, Ansel Hillmer, Rainer Hinz, Federico Turkheimer, Paolo Zanotti-Fregonara

**Affiliations:** 1 Edinburgh Imaging, University of Edinburgh, Edinburgh, EH16 4SB UK; 2 CEA, CNRS, MIRCen, Laboratoire des Maladies Neurodégénératives, Université Paris-Saclay, 92265 Fontenay-aux-Roses, France; 3 Departments of Radiology and Biomedical Imaging, Yale School of Medicine, New Haven, CT USA; 4 Departments of Psychiatry, Yale School of Medicine, New Haven, CT USA; 5 Yale PET Center, Yale School of Medicine, New Haven, CT USA; 6 Wolfson Molecular Imaging Centre, University of Manchester, Manchester, M20 3LJ UK; 7 Department of Neuroimaging, Institute of Psychiatry, Psychology and Neuroscience, Centre for Neuroimaging Sciences, King’s College London, De Crespigny Park, London, SE5 8AF UK; 8 MRC Centre for Neurodevelopmental Disorders, King’s College London, London, SE1 1UL UK; 9 Molecular Imaging Branch, National Institute of Mental Health, National Institutes of Health, Bethesda, MD USA

**Keywords:** Translocator protein 18 kDa (TSPO), Positron emission tomography (PET), Kinetic modeling, Inflammation

## Abstract

**Purpose:**

Translocator protein 18-kDa (TSPO) imaging with positron emission tomography (PET) is widely used in research studies of brain diseases that have a neuro-immune component. Quantification of TSPO PET images, however, is associated with several challenges, such as the lack of a reference region, a genetic polymorphism affecting the affinity of the ligand for TSPO, and a strong TSPO signal in the endothelium of the brain vessels. These challenges have created an ongoing debate in the field about which type of quantification is most useful and whether there is an appropriate simplified model.

**Methods:**

This review focuses on the quantification of TSPO radioligands in the human brain. The various methods of quantification are summarized, including the gold standard of compartmental modeling with metabolite-corrected input function as well as various alternative models and non-invasive approaches. Their advantages and drawbacks are critically assessed.

**Results and conclusions:**

Researchers employing quantification methods for TSPO should understand the advantages and limitations associated with each method. Suggestions are given to help researchers choose between these viable alternative methods.

## Introduction

Neuroinflammation is the inflammatory response of the brain and spinal cord. Although the physiological processes that occur within the neuroinflammatory response depend on the neurological pathologies involved, one common component of the neuro-immune response is activation of glial cells, predominantly microglia [1]. The translocator protein 18 kDa (TSPO), while being expressed ubiquitously in the body, is used as a biomarker of neuroinflammation because its upregulation in inflammatory conditions is strongly localized to glial cells and macrophages [2]. Over the years, a large number of positron emission tomography (PET) radioligands targeting TSPO have been developed. The prototypical radioligand for TSPO is [^11^C]-(*R*)-PK11195, which was first used for human brain imaging in 1989 to study glioma [3] and was subsequently applied to a variety of neurological [4, 5] and peripheral pathologies [6]. However, [^11^C]-(*R*)-PK11195 is associated with low amounts of specific binding, and new radioligands with better binding properties have been developed [7]; these include [^11^C]-PBR28 [8], [^18^F]-DPA-714 [9], and [^11^C]-ER176 [10].

The challenges associated with quantification are related to the biology of TSPO itself as well as to the properties of TSPO radioligands [11]. First, TSPO is present not only in glial cells of the brain parenchyma but also in the neurovascular unit [11], including endothelial cells, smooth muscle cells, and red blood cells [12, 13]. Second, TSPO has a genetic polymorphism (rs6971; Ala147Thr) that conveys different affinity profiles for TSPO radioligands—high-affinity, mixed-affinity, or low-affinity binders (HABs, MABs, and LABs, respectively) [14]; this requires that individuals be genotyped in order to stratify PET data, adding an extra blood test and analysis step. Third, the plasma free fraction (*f*_P_) of ligand available to enter the brain and bind to TSPO is very low for most ligands and can be difficult to measure [11]. Fourth, peripheral immune response can affect ligand response in blood and plasma as well as brain neuro-immune response. Indeed, the relationship between TSPO expression in the brain and levels of peripheral cytokines is an important topic of research. Finally, absolute quantification of TSPO requires invasive arterial blood sampling, which is time-consuming, costly, requires specialized equipment and personnel, and may not be well-tolerated, especially by patient populations. Taken together, these methodological challenges have created ongoing debate regarding the best way to quantify TSPO radioligands, particularly with regard to issues such as which kinetic model to use for analysis, whether an appropriate simplified model can be applied, and whether *f*_P_ should be included in the analysis.

This manuscript reviews the different quantification approaches and parameter estimation methods for TSPO radioligands, including (1) quantification of receptor density (*V*_T_) using the “gold standard” of full compartmental modeling and metabolite-corrected input function, with and without an additional compartment representing the binding of TSPO in the endothelium of blood vessels; (2) quantification of the non-displaceable fraction (*V*_ND_), and the strategies to measure *V*_ND_ without a blockade with pharmacological agents; and (3) quantification of the input function without placing an arterial catheter, which include techniques such as image-derived or population-derived input function, or the use of various types of reference regions. Finally, the manuscript also reviews problems linked to estimating *f*_P_.

The review aims to help researchers better understand the problems related to quantification of TSPO with PET and the advantages and drawbacks associated with the different published techniques.

## Compartmental modeling

### Classical compartmental modeling

**Fig. 1:**
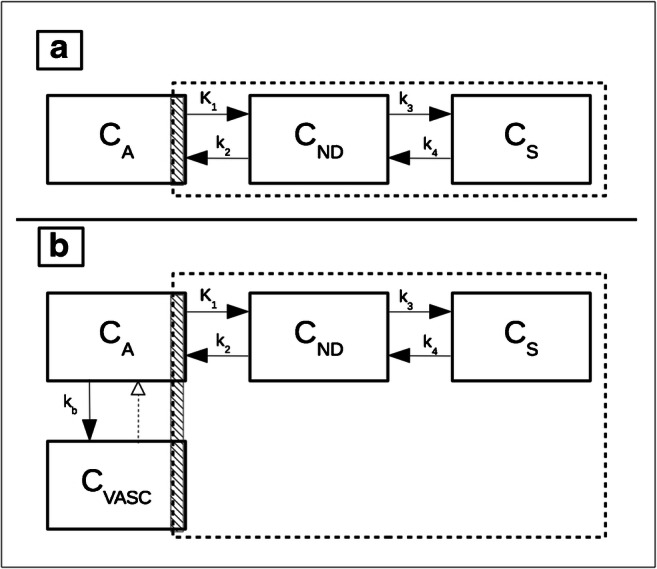
Compartmental models commonly used for parameter estimation of translocator protein 18-kDa (TSPO) radioligands when arterial input function (AIF) is available. **a** The two-tissue compartment model (2TCM). **b** The 2TCM with an extra compartment representing the radioligand specifically bound to vascular TSPO (2TCM-1K), as proposed by Rizzo and colleagues [15]. Each compartment represents a pool of radioligand concentration. *K*_1_ to *k*_4_ represent the rate constants between the compartments, and *k*_b_ represents the rate of binding to vascular TSPO. The dotted line indicates the concentrations captured by the PET scan and the hatched area indicates the vascular fraction. Abbreviations: *C*_*A*_, radioligand free in arterial plasma; *C*_*ND*_, non-displaceable ligand made up of free and nonspecific binding; *C*_*S*_, specifically bound ligand; *C*_*VASC*_, specifically bound ligand to vascular TSPO

Full compartmental modeling using a metabolite-corrected arterial input function (AIF) is considered the gold standard quantification method for PET data. For TSPO, it was first employed in [^11^C]-(*R*)-PK11195 studies using a reversible two-tissue compartment model (2TCM) (Fig. 1) [16]. The 2TCM includes compartments that account for radioactivity concentration of non-displaceable radioligand as well as specifically bound radioligand in brain tissue. Across all TSPO PET imaging studies, reversible 2TCM has been the most commonly applied model because it is usually preferred by fitting criteria [17–24].

The 2TCM is used to estimate kinetic rate constants (*K*_1_–*k*_4_) and macro-parameters that are made up of a combination of these constants [25]. The most often reported parameters for TSPO ligand binding are volume of distribution (*V*_*T*_ = (*K*_1_/*k*_2_)*(1 + *k*_3_/*k*_4_)) and binding potential (BP_ND_ = *k*_3_/*k*_4_). *V*_T_ includes specifically bound radioligand (*V*_S_) and free plus non-specifically bound or non-displaceable radioligand (*V*_ND_), where *V*_T_ = *V*_S_ + *V*_ND_. BP_ND_ is a composite measure of the affinity of the radioligand for the target and the density of the available target in vivo. BP_ND_ estimated from compartmental modeling alone is often unstable and therefore is generally only reported if a reference region or estimate of *V*_ND_ is available (BP_ND_ = (*V*_T_ − *V*_ND_)/*V*_ND_). For a complete list of kinetic parameters and their definitions, please see Innis et al. [25].

### Accounting for vascular TSPO via compartmental modeling

Histological data show that TSPO is expressed not only in activated glial cells but also in the vasculature (endothelial and smooth muscle cells) [26–31] to a degree that depends on tissue type and pathological status. The proximity of free radioligand in plasma to TSPO sites in the vasculature compared to TSPO sites in the brain parenchyma conveys different binding kinetics. Therefore, a compartmental model with only one specific binding compartment may not be adequate [11], and models accounting for TSPO binding in the vasculature have been proposed [15, 26].

Rizzo and colleagues introduced an additional irreversible compartment to the 2TCM for [^11^C]-PBR28, known as 2TCM-1K [15] (Fig. 1b). When the model was applied to PET data from healthy volunteers, parsimony criteria showed that 2TCM-1K described the data better than the standard 2TCM. In addition, TSPO mRNA levels from the Allen Human Brain Atlas [32] correlated better with *V*_T_ estimated from the 2TCM-1K than from the standard 2TCM. TSPO mRNA levels also correlated, albeit more weakly, with regional parameters related to vascular binding (*K*_b_) estimates from 2TCM-1K. Wimberley and colleagues largely reproduced these findings with [^18^F]-DPA714 in healthy volunteers and reported that 2TCM-1K fitted the data better than conventional 2TCM; they also found that TSPO mRNA levels strongly correlated not only with *K*_b_ but also with mRNA markers of endothelial cells [33]. This suggests that, in healthy brains, the regional variation of vascular TSPO appears to be stronger than the regional variation of TSPO expressed in glial cells (potentially due to differences in vascular density). Subsequently, Rizzo and colleagues extended these results by comparing models with and without the vasculature component for three TSPO radioligands with different affinities—[^11^C]-(*R*)-PK11195 had the lowest affinity, [^18^F]-DPA714 had middle affinity, and [^11^C]-PBR28 had the highest affinity. They found that models that accounted for vascular binding were preferred for all three ligands using the parsimony criteria [34]. Notably, these findings showed that the *K*_b_ parameter was related to ligand affinity (i.e., *K*_b_ for [^11^C]-(*R*)-PK11195 < [^18^F]-DPA714 < [^11^C]-PBR28). Finally, a study using [^11^C]-PBR28 and partial pharmacological blockade showed that 2TCM-1K described the data better than the 2TCM in two cohorts of young and old healthy volunteers, respectively. This model choice was supported by ex vivo evidence of endothelial TSPO, which showed that TSPO-positive vessels accounted for 30% of vascular volume in cortical and white matter [29]. In studies where vascular TSPO may change and thus mask parenchymal TSPO changes, both models should be tested, and the reliability of the parameter estimates should be evaluated. Appropriate simulations should be conducted to explore the ability of the model to separate vascular from parenchymal TSPO. In addition, changes in vascular and parenchymal TSPO binding as estimated by the 2TCM and 2TCM-1K models in vivo should be correlated to immunohistological data in patients with underlying pathologies.

### Limitations of compartmental models that include vascular uptake

The question of whether 2TCM or 2TCM-1K is the “best” model for investigating TSPO radioligands remains an unresolved topic within the PET imaging field. Indeed, multiple reports implementing analyses of both models indicate a preference for 2TCM over 2TCM-1K, in contrast to the studies discussed above. This preference has been noted for both [^11^C]-DPA713 scans acquired in patients with recent-onset schizophrenia [35] as well as [^11^C]-PBR28 scans conducted under both baseline conditions and after an endotoxin stimulus [36]. One may speculate that the 2TCM-1K may be preferable when the density of TSPO in the endothelium is relatively high compared to that in the glia, such as in healthy subjects, and when vascular manifestations are expected to play a prominent role in the disease. Conversely, the conventional 2TCM may be preferred in diseases where the density of parenchymal TSPO is increased more than that of the vascular TSPO. In either case, it is necessary to analyze the goodness of fit parameters and the stability of the parameter estimates.

Interestingly, 2TCM-1K assumes an irreversible binding to the vascular compartment even though TSPO radioligands are clearly reversible in the parenchyma. Use of an irreversible compartment is necessary given the difficulties associated with reliably estimating more parameters via a single-injection experiment. Indeed, additional parameters render the model parameter estimates less stable, and whether it is truly possible to separate different binding parameters remains controversial. This was demonstrated in the work of Hagens and colleagues [37], where parameter estimates were deemed too unreliable even though 2TCM-1K gave a better result in terms of fitting criteria than the 2TCM. In addition, while histology data clearly show co-localization of TSPO with endothelial cells that can be altered in disease states, the field lacks pharmacological data to support the dramatically different binding kinetics of TSPO on endothelial cells implied by the extra compartment in the 2TCM-1K model. More parameters also make 2TCM-1K more sensitive to noise, which may contribute to some conflicting results as regards model choice. De-noising or noise-reduction techniques can increase the stability of the model and investigators’ ability to determine the best model [33]. One way to test the validity of 2TCM-1K could be with a multiple-injection study, which would allow the separation of more parameters and the quantification of the bias generated by the simpler use of an irreversible compartment. As the evidence reviewed above suggests, however, the field currently lacks consensus regarding the optimal kinetic model to use when quantifying TSPO. Models that include a vascular compartment can allow separation of binding parameters related to vascular and parenchymal TSPO but the identification of the individual parameters will be less robust. The advantages and limitations of the different models, reviewed above, should be considered when choosing an analytic method.

## Estimation of *V*_ND_

As noted above, *V*_T_ encompasses both *V*_S_ and *V*_ND_. PET studies typically seek to detect group differences in specific signal (i.e., *V*_S_). *V*_T_ traditionally provides the most stable outcome measure but usually underestimates the true difference in *V*_S_. Furthermore, such an approach assumes that group differences in *V*_T_ stem from group differences in *V*_S_ and *V*_ND_ values that are comparable across groups or individuals. However, this may not be true due, for example, to metabolic differences in the pathological brain. *V*_ND_ is obtained through pharmacological blockade to generate an occupancy plot. Because pharmacological blockade is not a viable option for regular clinical protocols, two approaches that estimate *V*_ND_ of TSPO radioligands without pharmacological blockade have been proposed: (1) the polymorphism plot [22] and (2) the simultaneous estimation of *V*_ND_ (SIME) [38].

### Polymorphism plot

The polymorphism plot [22] estimates *V*_ND_ at the *population* level, so that population differences in *V*_ND_ (between healthy and pathological cohorts) can be removed from group comparisons. The approach leverages the two-site binding pattern of MAB tissue, assuming equal expression of high-affinity and low-affinity binding sites [39] to generate the following relationship:

1 $$ {V}_T^{\mathrm{HAB}}-{V}_T^{\mathrm{MAB}}=\Delta  \bullet \left({V}_T^{\mathrm{HAB}}-{V}_{\mathrm{ND}}\right) $$

where *Δ* is related to the ratio of the binding potentials of low-affinity sites to that of high-affinity sites. This relationship is analogous to the classic graphical relationship used to calculate receptor occupancy [40, 41]. This plot was used to estimate plausible *V*_ND_ values for [^18^F]-DPA714 [21] as well as for [^18^F]-PBR111, comparable to *V*_ND_ values estimated using the 2TCM [22]. However, application to data acquired with [^11^C]-ER176 yielded poor results, likely because the *V*_T_ of MABs is only slightly smaller than that of HABs [42, 43]. To our knowledge, this approach has not been applied to studies comparing group differences between populations. If such an approach were implemented, separate polymorphism plots for each study group would be required to account for possible group differences in *V*_T_ (and potentially *V*_ND_), although it remains unclear whether the polymorphism plot would yield statistical improvements (i.e., increase effect size).

### Simultaneous estimation of *V*_ND_ (SIME)

SIME estimates *V*_ND_ at the *individual* level. *V*_ND_ is obtained using data from multiple regions and coupling parameters across all regions. The SIME approach first assumes that *V*_ND_ is uniform throughout the brain, which is a standard assumption in modeling PET neuroimaging data (e.g., in reference region approaches). In the operational 2TCM equation, SIME replaces *K*_1_ with *k*_2_ *V*_ND_. Optimization of the cost function is then performed simultaneously on all the brain regions, in contrast to conventional independent optimization for each distinct brain region. Because *V*_ND_ is assumed to be uniform across regions, this parameter coupling reduces the number of estimated parameters from 4*r* to 3*r* + 1 (where *r* is the number of brain regions analyzed), improving micro-parameter estimates enough to reliably estimate both *V*_ND_ and *V*_S_ [38, 44].

Both simulations and human PET scan data have been used to evaluate SIME performance for [^11^C]-PBR28 [45]. Simulations demonstrated unbiased estimation of *V*_ND_ with reasonable precision, and human data suggested good test-retest variability [45]. Human studies with [^11^C]-PBR28 scans acquired after pharmacological blockade with the selective TSPO agonist XBD173 confirmed good agreement in *V*_ND_ estimation with conventional analysis approaches, although data were acquired only in HABs [41, 46]. Interestingly, different *V*_ND_ values were reported for MABs. If this finding is confirmed by blocking experiments, it would indicate that the rs6971 genotype may also affect *V*_ND_. When the SIME approach was used with [^11^C]-PBR28 to analyze data from Alzheimer’s disease patients and age-matched controls, *V*_ND_ values were found to be comparable between groups, and effect sizes calculated with *BP*_ND_ were larger than effect sizes calculated from conventional *V*_T_ estimates [44]. Although these studies demonstrate the statistical benefits of using BP_ND_ as an outcome measure over *V*_T_, it should be noted that the reliability of *BP*_ND_ is poor because uncertainty from both *V*_S_ and *V*_ND_ estimates is incorporated when calculating this outcome measure. Thus, *V*_S_ is the recommended outcome measure for use with SIME [45].

## Non-invasive input functions

The main practical obstacle to performing full quantitative PET studies is the need for an AIF. Although the rate of serious complications is exceedingly small in expert hands [47], placing a catheter in the radial artery and measuring the concentration of parent and radiometabolites in plasma are complex procedures that involve specialized personnel and equipment, require careful logistical planning, and increase the cost of PET scans considerably. These considerations have prompted the search for alternative methods of obtaining non-invasive proxy measures of the input function; some of the alternative approaches applied to TSPO PET imaging include image-derived input function (IDIF), population-based input function (PBIF), and SIME using a PBIF.

### IDIF

IDIF obtains the input function directly from a blood pool visible on PET images acquired dynamically. Even without considering the challenges posed by correcting partial volume effects in small brain vessels [48], the main obstacle to using IDIF in TSPO studies is the presence of radiolabeled metabolites in blood. All currently available TSPO ligands result in radiolabeled metabolites, and because PET scanners cannot distinguish photons emitted by the parent compound from those emitted by its radiometabolites, only the radioactivity concentration of whole blood can be obtained from the images. To perform radiometabolite correction, at least a small number of arterial blood samples are necessary, thus defeating the primary purpose of avoiding arterial catheterization. Mabrouk and colleagues calculated IDIF from the carotid arteries of individuals injected with the TSPO ligand [^18^F]-FEPPA, obtained with a modified independent component analysis algorithm [49]. However, despite sophisticated analyses, arterial blood was still necessary to reduce the variability and errors generated by the IDIF algorithm, to calculate the radiometabolites and the blood-to-plasma ratio, and to calibrate the blood curve. Zanotti-Fregonara and colleagues [50] used a high-resolution tomograph to calculate IDIF in [^11^C]-PBR28 brain scans from the carotid artery. The IDIFs remained very inaccurate even after scaling with blood samples, probably due to the difficulties associated with estimating the rapid peak and low concentrations of parent radioligand after the peak from the noisy voxels.

### PBIF

PBIF relies on the assumption that the shape of the bolus is constant among individuals and that only its amplitude changes. Thus, a predetermined template curve is scaled at the right amplitude using individual scaling factors, most often one or more blood samples. As with IDIF, this is as invasive as taking the full input function and necessarily yields less accurate results [51]. Mabrouk and colleagues evaluated the accuracy of a PBIF scaled with one arterial sample on a dataset of patients imaged with the TSPO ligand [^18^F]-FEPPA and found that PBIF increased the variability of the measurements without reducing invasiveness [52].

### SIME using PBIF

The SIME method, described above, can also be applied using a population-based input function to estimate BP_ND_ [44]. With this method, *V*_T_ and *V*_ND_ are simultaneously estimated for all regions—as in the SIME with AIF—but the AIF is replaced with a PBIF (no scaling). The error that comes from an inaccurate amplitude of the PBIF is assumed to be present in both *V*_T_ and *V*_ND_ estimates equally; thus, it is thought to cancel out in the calculation of the outcome parameter BP_ND_ ((α*V_T_-α*V_ND_)/α*V_ND_ where “α” is the error term induced by the difference in the amplitude of the PBIF compared to the true AIF). However, this technique has been described in only a single study that used [^11^C]-PBR28 [44] and has not been used with other TSPO ligands. The study examined a population of healthy volunteers and patients with Alzheimer’s disease and found good correlation between regional BP_ND_ measurements estimated using SIME with AIF and SIME with PBIF. As with the standard PBIF described above, the main assumption of this method is that the shape of the PBIF is identical between participants, though this will not always be the case, especially because pathology or other factors can alter metabolism.

## Reference and pseudo-reference regions

Given the limitations of non-invasively estimating the plasma concentration in blood vessels (i.e., the AIF), quantification has been attempted using a reference or pseudo-reference region. A reference region is defined as a region devoid of the target receptor but with a similar non-displaceable ligand profile (i.e., *K*_1_/*k*_2_ should be the same for the target and the reference region). However, because TSPO is ubiquitously expressed in the brain, a proper reference region does not exist [11]. As a result, the reference region for TSPO should be more properly referred to as a pseudo-reference region, defined as a region that contains the target under study but where its concentration does not change during disease.

Using a (pseudo)reference region is more accurate than measuring the simple concentration of radioactivity (such as that measured with standardized uptake value (SUV)), which may be influenced by cerebral blood flow and peripheral changes. When using another region of the same brain as a reference region, partial volume effects are less important than those affecting radioactivity concentrations in the vessels because the brain regions are usually bigger. In addition, radiometabolites and *f*_P_ are implicitly accounted for, thus reducing variability in the outcome measurements. This may increase the sensitivity of the study, as shown by Lyoo and colleagues, who were able to identify an additional pathological region in Alzheimer’s disease patients when using a reference region approach compared to full kinetic modeling [53].

To estimate TSPO binding parameters, uptake in the target regions can be normalized to the activity of the (pseudo)reference region using a simple SUV ratio (SUVr), or the reference curve can be used in a kinetic model such as the simplified reference tissue model (SRTM) [52, 54, 55] or graphical Logan reference plot [9]. These approaches have been used in clinical studies that used TSPO images to explore a variety of disorders, including Alzheimer’s disease [53, 55, 56], Parkinson’s disease [57], amyotrophic lateral sclerosis [58], psychosis [59], and glioma [60].

It should be noted that the properties of the appropriate (pseudo)reference region, such as the absence of target change under disease, should be demonstrated beforehand. For instance, the use of the cerebellum as a pseudo-reference region to quantify TSPO binding in Alzheimer’s disease patients was justified by histological proof that this region is largely spared from inflammatory changes occurring over the course of the disease and by previous confirmation with full kinetic modeling that its binding potential did not change in the populations under study [53].

Importantly, when using the SUVr, validating the appropriate timing of the measurement is critical; the ratio should ideally be taken when, and if, radioligand concentrations reach a relatively stable transient equilibrium. For this reason, previous validation against plasma input models to define the optimal time window is required. This point is particularly important when the reference region used is the white matter. Because the gray and white matter exhibit differences in radioligand kinetics, a transient equilibrium in gray matter regions may be reached at a different time point than with white matter.

The cerebellum, used as whole or gray or white matter, has been the most frequently used anatomical (pseudo)reference region for TSPO ligands, even though the presence of specific TSPO binding in this region has been demonstrated by several studies. For instance, in a human TSPO blocking study that used the radioligand [^11^C]-PBR28 antagonist XBD173 in healthy volunteers, Owen and colleagues found that about half of the *V*_T_ in the cerebellum was attributable to specific binding. Another study of 35 healthy volunteers found increased binding of [^11^C]-(*R*)-PK11195 associated with aging in cortical and sub-cortical regions as well as in the cerebellum [61]. Furthermore, even when cerebellar gray matter is a valid pseudo-reference region for a particular disease, it still contains non-negligible levels of TSPO binding. Therefore, BP_ND_ values derived from the SRTM or Logan reference plot using these regions will not reflect the “true” binding potential but rather a pseudo-binding potential. For instance, Plavén-Sigray and colleagues [62] reported that [^11^C]-*(R)*-PK11195 BP_ND_ values from the 2TCM using plasma input function were much higher than pseudo BP_ND_ values from the SRTM with cerebellum as the pseudo-reference region. In addition, signal in the cerebellum may, in some cases, be higher than that in other regions [63], and the BP_ND_ value will be negative, which is physiologically meaningless. To avoid negative results in these cases, some researchers choose to present results in terms of DVR (distribution volume ratio = BP_ND_ + 1) instead of BP_ND_.

The occipital cortex has also been used for normalization using [^11^C]-PBR28, specifically in subjects with chronic low back pain, amyotrophic lateral sclerosis [64], and fibromyalgia [65]. The pathological regions that were identified by using a (pseudo)reference region were similar to those identified by *V*_T_ estimated with compartmental modeling, but the SUVr was poorly correlated with *V*_T_ values [64].

In some studies, radioactivity concentrations are normalized to the value of the whole brain. This is a straightforward approach when there is a localized neuroinflammatory response and can be useful for detecting regional differences. For instance, after whole brain normalization, Zurcher and colleagues demonstrated increased TSPO binding in the motor cortex of patients with amyotrophic lateral sclerosis compared to controls [66]. Furthermore, Loggia and colleagues showed statistically significant increases in SUVr for multiple brain regions in chronic pain patients [67]. Nevertheless, using the whole brain as the reference made this approach insensitive to widespread or global effects, be it from disease [68] or pharmacological challenge [69]. In addition, because target brain regions themselves comprise some fraction of the whole brain, the numerator and denominator in the ratio are correlated, thus minimizing biological variability in the data [70]. Finally, whole-brain ratios are sensitive to possible differences in both gray and white matter uptake.

### Simplified reference tissue models with vascular component

Similar to the compartmental model with vascular component, a (pseudo) reference region model with a TSPO vascular binding component (SRTMv) was proposed in order to remove the confounding vascular component, especially in diseases where the vasculature is affected [26]. The SRTMv is a modified version of the SRTM that incorporates a blood volume parameter that modulates the signal from the carotids. The carotid curve includes radioligand binding to the vasculature and blood. SRTMv should be considered preferentially in cases where there are potential vascular and blood-brain barrier changes, such as in Alzheimer’s disease and dementia. To date, SRTMv has only been used with [^11^C]-(*R*)-PK11195 and is generally preferred to the standard SRTM because it is better able to discriminate between groups [34, 71, 72]. A recent test-retest study, however, found that estimates using SRTMv did not correlate with those derived from 2TCM, and that test-retest results were poor [62].

### Clustering for reference region extraction

A data-driven approach was developed for reference region quantification that defines the reference kinetics rather than using an anatomical region. The first example in the literature dates from 1999, when Banati and colleagues [73] used a voxel clustering approach to find an appropriate reference curve for dynamic [^11^C]-(*R*)-PK11195 images. More recently, a supervised clustering approach (SVCA) was proposed by Turkheimer and colleagues [30] for [^11^C]-(*R*)-PK11195 (see [74] for a review) and optimized by Yaqub and colleagues [71]. The SVCA method has also been adapted and validated for [^18^F]-DPA714 PET, albeit on a limited number of subjects (*n* = 10), all of whom were healthy volunteers [75]. SVCA uses a set of predefined kinetic curves projected through a dynamic image, voxel by voxel, so that each voxel time-activity curve is broken into a linear sum of the predefined curves. The method then filters the image such that the voxels made up of at least 95% of the lowest binding curve are taken as the reference region. The theoretical advantage of this technique is that, because it operates at the higher spatial resolution of the voxels, it would be able to extract a purer reference tissue signal (and therefore less contaminated by signal from specifically bound ligand) than had been possible from the anatomically defined volume of interest. Nevertheless, the SVCA method depends on the scanner characteristics and the properties of the reconstructed PET images. For instance, one requirement is camera-specific training datasets (population databases with dynamic images from healthy participants and from those with brain disorders with identified locally raised TSPO binding). Plavén-Sigray and colleagues [62] compared three such databases of participants imaged with [^11^C]-(*R*)-PK11195 using three different PET cameras and found that, although SRTM with the whole cerebellum as reference region underestimated BP_ND_ compared to SVCA-SRTM, the SVCA-derived curve still seemed to be contaminated by specific binding. Similarly, Zanotti-Fregonara and colleagues [76] applied SVCA to a large dataset of participants imaged with [^11^C]-PBR28 [53] and found that the extracted reference curves of HABs were markedly higher than those of MABs. This may be partly explained by the higher level of TSPO binding at baseline in HABs, but also by contamination from TSPO binding affecting reference tissue input curve estimates.

Rizzo and colleagues [34] then investigated whether SVCA could be applied to dynamic images from PET ligands with different affinities to TSPO [7]. They reported that sufficient contrast between gray matter and white matter was needed in order for SVCA to successfully partition the data. With increasing ligand affinity, vascular binding represented a higher proportion of total TSPO binding and, consequently, the brain tissue contrast decreased. By calculating the angles between the kinetic vectors of the gray and white matter for three ligands with different binding affinity, they showed that kinetic data from TSPO ligands with lower and medium affinity, such as [^11^C]-(*R*)-PK11195 and [^18^F]-DPA714, were more separable using SVCA than higher affinity, second-generation TSPO ligands such as [^11^C]-PBR28 [34].

## Plasma free fraction

*f*_P_ is the fraction of radioligand in plasma that is not bound to plasma proteins at equilibrium [25]. It is typically measured with ultrafiltration cartridges that are centrifuged to separate a plasma sample into free and plasma protein–bound fractions. In theory, *V*_T_ should be corrected by *f*_P_ (i.e., *V*_T_/*f*_P_), because only the *unbound* radioligand concentration in plasma is available to enter the tissue. Unfortunately, low *f*_P_ values can be unreliable due to the Poisson nature of these measurements, resulting in diminished precision with lower counting statistics. For example, an [^11^C]-PBR28 study in patients with alcohol use disorder and healthy controls that used *V*_T_/*f*_P_ as an outcome measure reported that the rs6971 genotype alleles were not significantly different due to the high uncertainty introduced with this measure [68]. In the case of TSPO radioligands, *f*_P_ tends to be quite low (generally <5%) [7]. As a result, many researchers choose *V*_T_ as a more stable outcome measure, especially if *f*_P_ is not expected to change between healthy volunteers and patients or does not statistically differ once the measurements are acquired. However, if the disease is expected to change *f*_P_, or if the study involves the administration of drugs, this measurement may become critical.

Notably, Cumming and colleagues [7] found that for TSPO ligands, low *f*_P_ values corresponded to high perfusion rates (~50%) for all radioligands (except [^18^F]-GE180, which cannot cross the blood-brain barrier in humans [77]). This suggests that plasma protein binding for these ligands is generally reversible and that significant fractions of these ligands, even if bound to plasma proteins, are nevertheless released into plasma by passing through the capillaries [7]. Obviously, changes to plasma proteins would affect these dynamics; as a result, the use of compartmental models and the accurate estimation of plasma-to-tissue transfer constants (generally indicated as *K*_1_) become necessary steps for accurately interpreting these data. The confound due to potential changes in *f*_*p*_ due to disease or drug interaction should be considered when interpreting parameter estimates, particularly *K*_1_ and macro-parameters that include *K*_1_, such as *V*_T_.

## Conclusion

Full compartmental modeling with AIF and 2TCM is the gold standard for quantifying TSPO PET radioligands. As noted above, some recent improvements and simplifications in pharmacokinetic modeling have been suggested, such as introducing a vascular component to account for TSPO vascular binding, or the SUVr, SRTM, and SVCA approaches using anatomical or extracted reference regions. It is crucial that researchers employing these methods understand the advantages and limitations associated with each one, and how methods for different ligands have been validated in order to select the most appropriate analytic methods for their studies.

From this review of the methods presented and the validation of the different methods, the following suggestions for quantification of TSPO PET studies are made:

1. TSPO PET should preferably be acquired with a metabolite-corrected input function, especially if global brain effects are expected. A pseudo-reference region can be used only if it has previously been validated for the disease and the radioligand under study. The whole brain can be used as a pseudo-reference region when only regional differences are investigated. In addition, simplified non-invasive methods must be validated beforehand with a metabolite-corrected input function.
2. Non-invasive input functions, such as IDIF or PBIF, should be avoided. Non-invasive SIME requires further validation before its use can be recommended.
3. SVCA should be used preferentially for [^11^C]-(*R*)-PK11195 PET studies. More extensive validation is warranted for radioligands with higher specific binding, especially by replicating clinical protocols that include both healthy volunteers and patients and where images have been quantified with full kinetic modeling.
4. The use of compartmental models with an additional vascular compartment for TSPO PET has strengths and weaknesses that need to be considered. Adding a vascular compartment allows differentiation of changes in the outcome parameters due to vascular binding and parenchymal uptake, but these advantages occur at the price of less robust identification of the individual parameters.
5. Although methods for estimating *V*_ND_ (polymorphism plot, SIME) may result in improved parameter estimation compared to conventional quantification methods, discrepancies across studies suggest that further investigation and validation are needed.
6. Correcting *V*_T_ for the *f*_*p*_ increases the variability of measurements. Therefore, this correction should preferably be used when a variation in *f*_*p*_ is expected, such as when a blocking agent is given, as well as when statistical comparison between two groups shows significant average difference in *f*_*p*_.
